# Association between gestational weight gain and preterm birth and post-term birth: a longitudinal study from the National Vital Statistics System database

**DOI:** 10.1186/s12887-023-03951-0

**Published:** 2023-03-20

**Authors:** Yifang Zhu, Jiani Zhang, Qiaoyu Li, Min Lin

**Affiliations:** Department of Pediatrics, The Second Affiliated Hospital of Fujian Medical University, No.34 Zhongshan North Road, Licheng District, Quanzhou, 362000 P.R. China

**Keywords:** Preterm birth, Post-term birth, Gestational weight gain, Body mass index

## Abstract

**Background:**

To evaluate the association between gestational weight gain (GWG) and preterm birth and post-term birth.

**Methods:**

This longitudinal-based research studied singleton pregnant women from the National Vital Statistics System (NVSS) (2019). Total GWG (kg) was converted to gestational age-standardized z scores. The z-scores of GWG were divided into four categories according to the quartile of GWG, and the quantile 2 interval was used as the reference for the analysis. Univariate and multivariate logistic regression analyses were performed to investigate the association between GWG and preterm birth, post-term birth, and total adverse outcome (preterm birth + post-term birth). Subgroup analysis stratified by pre-pregnancy body mass index (BMI) was used to estimate associations between z-scores and outcomes.

**Results:**

Of the 3,100,122 women, preterm birth occurred in 9.45% (292,857) population, with post-term birth accounting for 4.54% (140,851). The results demonstrated that low GWG z-score [odds ratio (OR): 1.04, 95% confidence interval (CI): 1.03 to 1.05, *P* < 0.001], and higher GWG z-scores (quantile 3: OR: 1.42, 95% CI: 1.41 to 1.44, *P* < 0.001; quantile 4: OR: 2.79, 95% CI: 2.76 to 2.82, *P* < 0.001) were positively associated with preterm birth. Low GWG z-score (OR: 1.18, 95% CI: 1.16 to 1.19, *P* < 0.001) was positively associated with an increased risk of post-term birth. However, higher GWG z-scores (quantile 3: OR: 0.84, 95% CI: 0.83 to 0.85, *P* < 0.001; quantile 4: 0.59, 95% CI: 0.58 to 0.60, *P* < 0.001) was associated with a decreased risk of post-term birth. In addition, low GWG z-score and higher GWG z-scores were related to total adverse outcome. A subgroup analysis demonstrated that pre-pregnancy BMI, low GWG z-score was associated with a decreased risk of preterm birth among BMI-obesity women (OR: 0.96, 95% CI: 0.94 to 0.98, *P* < 0.001).

**Conclusion:**

Our result suggests that the management of GWG may be an important strategy to reduce the number of preterm birth and post-term birth.

**Supplementary Information:**

The online version contains supplementary material available at 10.1186/s12887-023-03951-0.

## Background

Preterm birth is defined as birth before the completion of 37 weeks gestation, with one in 10 babies being born preterm, and every year, around 15 million babies are born preterm in the world, putting the global preterm birth rate at 11% [1, 2]. Post-term is defined as a pregnancy that has extended to or beyond 42 weeks (294 days) from the first day of the last normal menstrual period or 14 days beyond the best obstetric estimate of the date of delivery [3]. Preterm birth and post-term birth are both the cause of perinatal mortality and severe morbidity [4, 5], and impose a considerable burden on health, education, and social services, as well as on families and caregivers [6]. Given both preterm and post-term births are associated with unfavorable maternal and neonatal outcomes, the identification of modifiable risk factors is of great importance for the prevention of adverse outcomes from preterm birth and post-term birth.

Gestational weight gain (GWG) is necessary to ensure fetal health [7]. GWG reflects a variety of characteristics, including the accumulation of maternal fat, fluid swelling, and the growth of the fetus, placenta, and uterus [8]. Nevertheless, studies have found that excessive or insufficient GWG was associated with adverse outcomes [9–12]. Previous studies have identified an association between GWG and the risk of preterm birth [13, 14]. However, there is a limited study reporting the effect of GWG on post-term birth. Moreover, it is of particular importance to understand the relationships of GWG with outcomes of preterm and post-term birth combined, and to develop a reasonable pregnancy weight control plan to help to reduce the likelihood of both preterm birth and post-term birth.

The purpose of this study was to examine the associations between GWG and preterm birth, post-term birth, and the combined outcome of preterm and post-term birth; investigate the effect of GWG on preterm birth, post-term birth, and the combined outcome of preterm and post-term birth among different pregnancy body mass index (BMI) women.

## Methods

### Study design and population

This longitudinal study recruited pregnant women from the National Vital Statistics System (NVSS) (2019), which is a U.S. population-based retrospective cohort study from 50 States and the District of Columbia [15]. The NVSS is a major cooperative effort between the U.S. Centers for Disease Control and Prevention (CDC) and all U.S. states, which gathers information on maternal exposures before and during pregnancy and infant outcomes at delivery using two uniform documents: a facility worksheet and a maternal worksheet. Detailed methods, quality control, and vital statistics can be found on the CDC website (https://www.cdc.gov/nchs/nvss/births.htm). We included singleton pregnant women aged 18 years or older. Women with pre-pregnancy hypertension or diabetes, height < 55 inches, gestation at < 22 weeks or > 44 weeks, fetal malformations, chromosomal disorders, and pregnant women who used assisted reproduction were excluded. The de-identified data are publicly available online, so the ethical board review of the corresponding author's institution is exempted.

### Definitions

Maternal GWG was classified by GWG z-score of standardized maternal weight gain in gestational age, which was calculated as: (observed total weight gain- mean week-specific weight gain)/standard deviation of week-specific weight gain, with week-specific means and standard deviations [16]. The z-scores of GWG were divided into four categories according to the quartile of GWG, and the quantile 2 interval was used as the reference for analysis. Quantile 1 was 0.284, the Median was 0.439, and Quantile 3 was 0.573 of the z-scores levels of GWG. The maternal GWG ranges for different gestational ages based on the calculation of z-scores are shown in the Supplementary Table 1. Gestational age was determined based on the last menstrual period [17].

We classified maternal pre-pregnancy BMI as underweight (BMI < 18.5 kg/m^2^), normal weight (18.5–24.9 kg/m^2^), overweight (25–29.9 kg/m^2^), and obesity (≥ 30 kg/m^2^) [18].

### Potential covariates

Potential covariates included the number of prenatal visits, maternal age at pregnancy, smoking before pregnancy, prior other terminations, previous preterm birth, multipara or not, maternal race, the special supplemental nutrition program for women, infants, and children (WIC) food during pregnancy, maternal education, smoking during pregnancy. Maternal race was divided into White, Black, Asian, and other. The maternal education level was divided into < 12 grade, high school or general educational development (GED), some college, bachelor or above. A multipara is a woman who has given birth to more than one child.

### Outcomes

Preterm birth referred to < 37 weeks of gestation, term birth was >  = 37–42 weeks of gestation, and post-term birth was defined as > 42 weeks of gestation [19].

### Statistical analysis

The normally distributed measurement data were expressed as mean +—standard deviation, and the one-way analysis of variance (ANOVA) was used for comparison between groups; abnormally distributed measurement data were described as median and quartile [M (Q_1_, Q_3_)], and the comparison between groups was conducted by Kruskal–Wallis test. The enumeration data were compared using the chi-square test or Fisher’s exact test, manifesting as cases and the constituent ratio (n (%)). For the handling of missing data, the simple deletion method was used to delete the cases with missing values, sensitivity analysis before and after data deletion was performed.

Comparison between groups showed the characteristics of the study population with preterm birth, term birth, and post-term birth. With preterm birth and post-term birth as outcomes, the incidence of preterm birth and post-term birth in different GWG z-scores were calculated. We combined the preterm birth and post-term birth as a new outcome (total adverse outcome), and calculated the incidence of total adverse outcome in different GWG z-scores. To determine which confounders required adjustment, directed acyclic graph (DAG) were drawn (Supplementary Fig. 1). Univariate multivariate logistic regression model was used to explore the effect of GWG (weight gain z-score) on different gestational age groups. Model 1 was an unadjusted model, model 2 adjusted for number of prenatal visits, maternal age at pregnancy, smoking before pregnancy, prior other terminations, previous preterm birth, multipara or not, maternal race, WIC food during pregnancy, maternal education, and smoking during pregnancy. Subgroup analysis was stratified by early-pregnancy BMI to investigate the effects of GWG z-scores on preterm birth and post-term birth, and total adverse outcome.

All statistical tests were two-sided and a p-value < 0.05 was considered statistically significant. All statistical analyses were completed using Statistical Analysis Software version 9.4 (SAS Institute Inc.).

## Results

### Characteristics of the study population

**Fig. 1:**
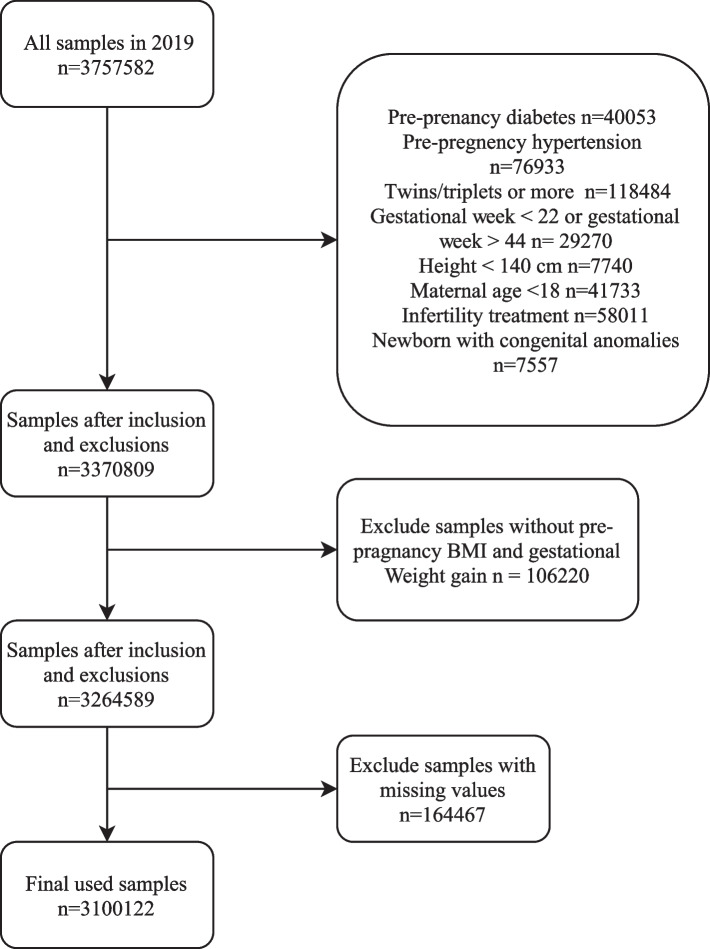
The flow diagram of participants selection

Following our exclusions, 3,100,122 women with live singleton births were included in the analysis (Fig. 1). Preterm birth occurred in 9.45% (292,857) population, with post-term birth accounting for 4.54% (140,851). In our study, 3.08% (95,608) women were underweight, 41.87% (1,297,882) normal weight, 27.23% (844,091) were overweight, and 27.82% (862,541) obese. Over half of our study was White (74.19%); Black, Asian, and other races constituted 15.21%, 6.68%, and 3.91% respectively. The mean GWG for women with preterm birth was 11.35 (7.26, 15.89) kg, and the mean GWG for women with post-term birth was 13.62 (9.08, 17.71) kg. Table 1 shows the baseline characteristics of the study population.

**Table 1:** Characteristics of the study population

Variables	Total (*n* = 3,100,122)	Preterm birth (*n* = 292,857)	Term birth (*n* = 2,666,414)	Post-term birth (*n* = 140,851)	Statistics	*P*
Maternal age at pregnancy, years, Mean ± SD	29.05 ± 5.60	29.11 ± 5.97	29.09 ± 5.56	28.07 ± 5.47	F = 2267.709	< 0.001
Maternal race, n (%)					χ^2^ = 11,577.64	< 0.001
White	2,300,082 (74.19)	199,429 (68.10)	1,993,838 (74.78)	106,815 (75.84)		
Black	471,532 (15.21)	63,396 (21.65)	387,968 (14.55)	20,168 (14.32)		
Asian	207,156 (6.68)	17,141 (5.85)	182,473 (6.84)	7542 (5.35)		
Others	121,352 (3.91)	12,891 (4.40)	102,135 (3.83)	6326 (4.49)		
Maternal education, n (%)					χ^2^ = 16,491.96	< 0.001
< 12 grade	347,915 (11.22)	44,010 (15.03)	285,328 (10.70)	18,577 (13.19)		
High school or GED	819,024 (26.42)	89,561 (30.58)	687,805 (25.80)	41,658 (29.58)		
Some college	884,826 (28.54)	84,866 (28.98)	758,856 (28.46)	41,104 (29.18)		
Bachelor or above	1,048,357 (33.82)	74,420 (25.41)	934,425 (35.04)	39,512 (28.05)		
Pre-pregnancy BMI, n (%)					χ^2^ = 2585.980	< 0.001
Underweight	95,608 (3.08)	10,951 (3.74)	80,502 (3.02)	4155 (2.95)		
Normal	1,297,882 (41.87)	113,921 (38.90)	1,127,107 (42.27)	56,854 (40.36)		
Overweight	844,091 (27.23)	77,880 (26.59)	728,318 (27.31)	37,893 (26.90)		
Obesity	862,541 (27.82)	90,105 (30.77)	730,487 (27.40)	41,949 (29.78)		
GWG, kg, M (Q_1_, Q_3_)	13.17 (9.08, 17.25)	11.35 (7.26, 15.89)	13.17 (9.08, 17.25)	13.62 (9.08, 17.71)	χ^2^ = 15,994.35#	< 0.001
GWG z-score, M (Q_1_, Q_3_)	0.44 (0.28, 0.57)	0.52 (0.35, 0.69)	0.44 (0.28, 0.57)	0.40 (0.23, 0.52)	χ^2^ = 43,068.24#	< 0.001
Smoking before pregnancy, n (%)					χ^2^ = 3930.086	< 0.001
No	2,862,009 (92.32)	262,842 (89.75)	2,471,714 (92.70)	127,453 (90.49)		
Yes	238,113 (7.68)	30,015 (10.25)	194,700 (7.30)	13,398 (9.51)		
Smoking during pregnancy, n (%)					χ^2^ = 4932.560	< 0.001
No	2,941,838 (94.89)	270,492 (92.36)	2,539,377 (95.24)	131,969 (93.69)		
Yes	158,284 (5.11)	22,365 (7.64)	127,037 (4.76)	8882 (6.31)		
Number of prenatal visits, M (Q_1_, Q_3_)	12.00 (9.00, 13.00)	10.00 (7.00, 12.00)	12.00 (10.00, 13.00)	12.00 (10.00, 14.00)	χ^2^ = 68,358.24#	< 0.001
Prior other terminations, n (%)					χ^2^ = 1352.827	< 0.001
No	2,258,968 (72.87)	205,929 (70.32)	1,947,264 (73.03)	105,775 (75.10)		
Yes	841,154 (27.13)	86,928 (29.68)	719,150 (26.97)	35,076 (24.90)		
History of preterm birth, n (%)					χ^2^ = 31,201.89	< 0.001
No	2,995,626 (96.63)	266,590 (91.03)	2,591,520 (97.19)	137,516 (97.63)		
Yes	104,496 (3.37)	26,267 (8.97)	74,894 (2.81)	3335 (2.37)		
WIC food during pregnancy, n (%)					χ^2^ = 5200.329	< 0.001
No	2,064,986 (66.61)	179,731 (61.37)	1,796,666 (67.38)	88,589 (62.90)		
Yes	1,035,136 (33.39)	113,126 (38.63)	869,748 (32.62)	52,262 (37.10)		
Gestational age, Mean ± SD	38.73 ± 2.01	34.38 ± 2.18	39.00 ± 1.11	42.58 ± 0.74	F = 2,563,966	< 0.001
Gender of newborn, n (%)					χ^2^ = 801.768	< 0.001
Female	1,513,848 (48.83)	136,304 (46.54)	1,306,395 (48.99)	71,149 (50.51)		
Male	1,586,274 (51.17)	156,553 (53.46)	1,360,019 (51.01)	69,702 (49.49)		
Multipara, n (%)					χ^2^ = 1769.685	< 0.001
No	1,178,778 (38.02)	104,300 (35.61)	1,015,028 (38.07)	59,450 (42.21)		
Yes	1,921,344 (61.98)	188,557 (64.39)	1,651,386 (61.93)	81,401 (57.79)		
Eclampsia, n (%)					χ^2^ = 4606.815	< 0.001
No	3,092,573 (99.76)	290,422 (99.17)	2,661,519 (99.82)	140,632 (99.84)		
Yes	7549 (0.24)	2435 (0.83)	4895 (0.18)	219 (0.16)		
Hypertension during pregnancy, n (%)					χ^2^ = 30,026.00	< 0.001
No	2,865,842 (92.44)	247,322 (84.45)	2,485,062 (93.20)	133,458 (94.75)		
Yes	234,280 (7.56)	45,535 (15.55)	181,352 (6.80)	7393 (5.25)		

*GED* general educational development, *BMI* body mass index, *GWG* gestational weight gain, *WIC* special supplemental nutrition program for women, infants, and children, *χ*^*2*^ chi-square test, *F* analysis of variance, *SD* standard deviation, *M* Median, *Q*_*1*_ 1st Quartile, *Q*_*3*_ 3rd Quartile

### Incidences of preterm birth, post-term birth, and total adverse outcome in different GWG z-score intervals

**Fig. 2:**
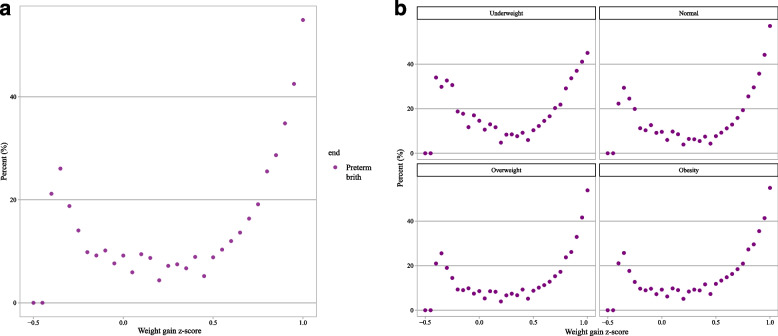
Incidences of preterm birth in different GWG z-score intervals; **a** total; **b** subgroup analysis of pre-pregnancy BMI

**Fig. 3:**
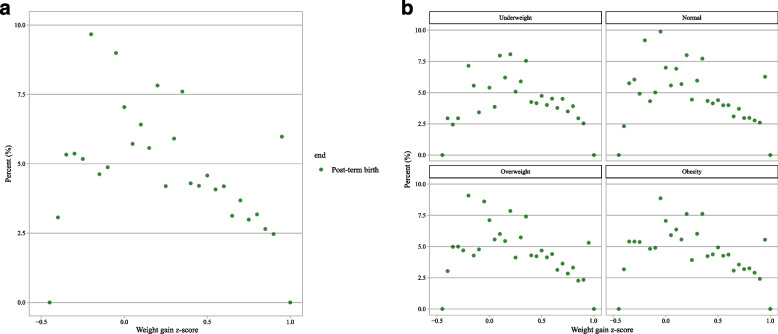
Incidences of post-term birth in different GWG z-score intervals; **a** total; **b** subgroup analysis of pre-pregnancy BMI

**Fig. 4:**
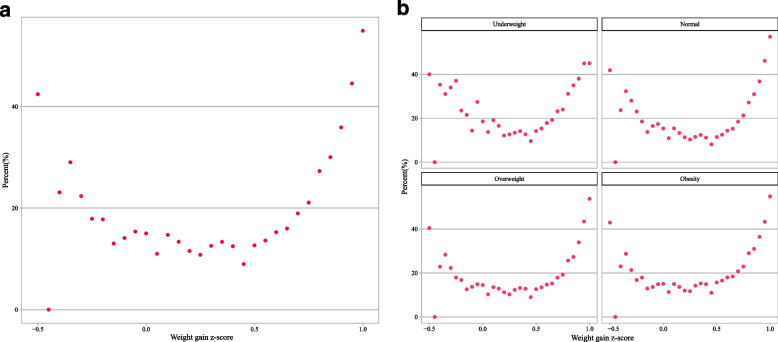
Incidences of total adverse outcome in different GWG z-score intervals; **a** total; **b** subgroup analysis of pre-pregnancy BMI

U-shaped relations have been observed between the GWG z-score and preterm birth. The relationship between GWG z-score and incidence of preterm birth is depicted in Fig. 2a and b. The results showed an approximately inverted U-shape between GWG z-score and post-term birth. The incidence of post-term birth in different GWG z-score intervals is depicted in Fig. 3a and b. Similar to the association between GWG z-score and preterm birth, the association of total GWG z-scores with total adverse outcome tended to be U-shaped. The relationship between GWG z-score and incidence of total adverse outcome is shown in Fig. 4a and b.

### Associations of GWG with preterm birth, post-term birth, and total adverse outcome

The result demonstrated that the weight-gain z-score in quantile 1 [odds ratio (OR): 1.04, 95% confidence interval (CI): 1.03 to 1.05, *P* < 0.001], quantile 3 (OR: 1.42, 95% CI: 1.41 to 1.44, *P* < 0.001), quantile 4 (OR: 2.79, 95% CI: 2.76 to 2.82, *P* < 0.001), and total weight-gain z-score (OR: 5.46, 95% CI: 5.37 to 5.55,* P* < 0.001) was associated with an increased risk of preterm birth. As for the post-term birth, the weight-gain z-score in quantile 1 was related to an increased risk of post-term birth (OR: 1.18, 95% CI: 1.16 to 1.19,*P* < 0.001). Nevertheless, weight-gain z-scores in 3 (OR: 0.84, 95% CI: 0.83 to 0.85, *P* < 0.001), quantile 4 (0.59, 95% CI: 0.58 to 0.60, *P* < 0.001), and total weight-gain z-score (OR: 0.49, 95% CI: 0.48 to 0.50,* P* < 0.001) related to a decreased risk of post-term birth. Concerning the total adverse outcome, weight-gain z-scores in quantile 1 (OR: 1.06, 95% CI: 1.05 to 1.07, *P* < 0.001), quantile 3 (OR: 1.17, 95% CI: 1.16 to 1.18, *P* < 0.001), quantile 4 (OR: 1.77, 95% CI: 1.75 to 1.78, *P* < 0.001), and total weight-gain z-score (OR: 2.31, 95% CI: 2.28 to 2.34, *P* < 0.001) were all associated with an increased risk of total adverse outcome. Associations of GWG with preterm birth, post-term birth, and total adverse outcome are presented in Table 2.

**Table 2:** Associations of GWG with preterm birth, post-term birth, and total adverse outcome

	Preterm birth				Post-term birth				Total adverse outcome			
	Model 1		Model 2		Model 1		Model 2		Model 1		Model 2	
Variables	OR (95% CI)	*P*	OR (95% CI)	*P*	OR (95% CI)	*P*	OR (95% CI)	*P*	OR (95% CI)	*P*	OR (95% CI)	*P*
Levels of weight-gain-for-gestational-age z-scores												
Quantile 1	1.24 (1.22–1.25)	< 0.001	1.04 (1.03–1.05)	< 0.001	1.19 (1.17–1.21)	< 0.001	1.18 (1.16–1.19)	< 0.001	1.19 (1.18–1.20)	< 0.001	1.06 (1.05–1.07)	< 0.001
Quantile 2	Ref		Ref		Ref		Ref		Ref		Ref	
Quantile 3	1.34 (1.32–1.35)	< 0.001	1.42 (1.41–1.44)	< 0.001	0.85 (0.84–0.86)	< 0.001	0.84 (0.83–0.85)	< 0.001	1.13 (1.12–1.14)	< 0.001	1.17 (1.16–1.18)	< 0.001
Quantile 4	2.68 (2.65–2.71)	< 0.001	2.79 (2.76–2.82)	< 0.001	0.62 (0.61–0.63)	< 0.001	0.59 (0.58–0.60)	< 0.001	1.77 (1.76–1.79)	< 0.001	1.77 (1.75–1.78)	< 0.001
Weight-gain-for-gestational-age z-scores	4.26 (4.19–4.33)	< 0.001	5.46 (5.37–5.55)	< 0.001	0.51 (0.50–0.52)	< 0.001	0.49 (0.48–0.50)	< 0.001	1.93 (1.90–1.95)	< 0.001	2.31 (2.28–2.34)	< 0.001

Model 1: unadjusted model; Model 2 adjusted for number of prenatal visits, maternal age at pregnancy, smoking before pregnancy, prior other terminations, previous preterm birth, multipara or not, maternal race, WIC food during pregnancy, maternal education, smoking during pregnancy, pre-pregnancy BMI

*GWG* gestational weight gain, *Ref* Reference, *OR* odds ratio, *CI* confidence interval

### Associations of GWG with preterm birth, post-term birth, and total adverse outcome in different pre-pregnancy BMI populations

Associations of GWG with preterm birth, post-term birth, and total adverse outcome in different pre-pregnancy BMI populations are shown in Table 3. Regarding preterm birth, weight-gain z-scores in quantile 1, quantile 3, quantile 4, and total weight-gain z-scores were all associated with an increased risk of preterm birth in women who were underweight, normal, and overweight. However, in women who were obese, the weight-gain z-score in quantile 1 was related to a lower risk of preterm birth (OR: 0.96, 95% CI: 0.94 to 0.98, *P* < 0.001) while weight-gain z-scores in quantile 2, in quantile 4, and total weight-gain z-scores were associated with a higher risk of preterm birth.

**Table 3:** Associations of GWG with preterm birth, post-term birth, and total adverse outcome in different pre-pregnancy BMI populations

Subgroups	OR (95% CI)	*P*	OR (95% CI)	*P*	OR (95%CI)	*P*
**Underweight** (*n* = 84,657)						
Levels of weight-gain-for-gestational-age z-scores						
Quantile 1	1.27 (1.16–1.38)	< 0.001	1.28 (1.16–1.42)	< 0.001	1.23 (1.15–1.31)	< 0.001
Quantile 2	Ref		Ref		Ref	
Quantile 3	1.47 (1.38–1.56)	< 0.001	0.85 (0.79–0.92)	< 0.001	1.23 (1.17–1.29)	< 0.001
Quantile 4	2.88 (2.72–3.06)	< 0.001	0.60 (0.55–0.65)	< 0.001	1.90 (1.81–1.99)	< 0.001
Weight-gain-for-gestational-age z-scores	11.77 (10.51–13.17)	< 0.001	0.31 (0.26–0.36)	< 0.001	4.30 (3.90–4.73)	< 0.001
**Normal** (*n* = 1,183,961)						
Levels of Weight-gain-for-gestational-age z-scores						
Quantile 1	1.17 (1.15–1.20)	< 0.001	1.19 (1.16–1.22)	< 0.001	1.13 (1.11–1.15)	< 0.001
Quantile 2	Ref		Ref		Ref	
Quantile 3	1.46 (1.43–1.49)	< 0.001	0.80 (0.78–0.82)	< 0.001	1.14 (1.12–1.16)	< 0.001
Quantile 4	3.07 (3.02–3.13)	< 0.001	0.56 (0.54–0.57)	< 0.001	1.78 (1.75–1.80)	< 0.001
Weight-gain-for-gestational-age z-scores	11.88 (11.49–12.28)	< 0.001	0.33 (0.32–0.34)	< 0.001	3.31 (3.22–3.40)	< 0.001
**Overweight** (*n* = 766,211)						
Levels of Weight-gain-for-gestational-age z-scores						
Quantile 1	1.00 (0.98–1.03)	0.978	1.18 (1.15–1.21)	< 0.001	1.04 (1.02–1.06)	< 0.001
Quantile 2	Ref		Ref		Ref	
Quantile 3	1.40 (1.37–1.44)	< 0.001	0.88 (0.85–0.90)	< 0.001	1.17 (1.15–1.20)	< 0.001
Quantile 4	2.64 (2.59–2.70)	< 0.001	0.62 (0.60–0.64)	< 0.001	1.72 (1.69–1.75)	< 0.001
Weight-gain-for-gestational-age z-scores	5.71 (5.53–5.90)	< 0.001	0.50 (0.48–0.52)	< 0.001	2.37 (2.31–2.43)	< 0.001
**Obesity** (*n* = 772,436)						
Levels of Weight-gain-for-gestational-age z-scores						
Quantile 1	0.96 (0.94–0.98)	< 0.001	1.18 (1.16–1.21)	< 0.001	1.02 (1.01–1.04)	0.011
Quantile 2	Ref		Ref		Ref	
Quantile 3	1.44 (1.41–1.47)	< 0.001	0.89 (0.86–0.92)	< 0.001	1.23 (1.20–1.25)	< 0.001
Quantile 4	2.55 (2.50–2.61)	< 0.001	0.62 (0.60–0.64)	< 0.001	1.78 (1.75–1.81)	< 0.001
Weight-gain-for-gestational-age z-scores	3.30 (3.23–3.38)	< 0.001	0.59 (0.58–0.61)	< 0.001	1.81 (1.78–1.85)	< 0.001

The model adjusted number of prenatal visits, maternal age at pregnancy, smoking before pregnancy, prior other terminations, previous preterm birth, multipara or not, maternal race, WIC food during pregnancy, maternal education, smoking during pregnancy

*GWG* gestational weight gain, *BMI* body mass index, *Ref* Reference, *OR* odds ratio, *CI* confidence interval

As for the post-term birth, weight-gain z-score in quantile 1 was related to an increased risk of post-term birth in women who were underweight (OR: 1.28, 95% CI: 1.16 to 1.42, *P* < 0.001), normal (OR: 1.19, 95% CI: 1.16 to 1.22, *P* < 0.001), overweight (OR: 1.18, 95% CI: 1.15 to 1.21,* P* < 0.001), and obesity (OR: 1.18, 5% CI: 1.16 to 1.21, *P* < 0.001), however, weight-gain z-scores in quantile 3, quantile 4, and total weight-gain z-scores were all associated with a decreased risk of post-term birth in women who were underweight, normal, overweight, and obesity.

In terms of total adverse outcome, weight-gain z-scores in quantile 1, quantile 3, quantile 4, and total weight-gain z-scores were all associated with total adverse outcome among underweight, normal, overweight, and obese women.

## Discussion

In this large population-based study of more than a million women with live singleton births in the U.S., we found that low GWG z-score and higher GWG z-scores were positively associated with preterm birth. A low GWG z-score was positively associated with an increased risk of post-term birth. However, higher GWG z-score (excessive GWG) was associated with a decreased risk of post-term birth. In addition, a low GWG z-score and higher GWG z-scores were related to total adverse outcome. When stratified by pre-pregnancy BMI, low GWG z-score was associated with a decreased t risk of preterm birth among BMI-obesity women.

In this study, we found that low GWG z-score and higher GWG z-scores were positively associated with preterm birth. In accordance with our findings, a study by Santos et al. reported that both lower and higher total gestational weight gain z-scores were associated with a higher risk of preterm birth [20]. A study compared GWG z-scores and traditional weight gain measures in relation to perinatal outcomes found that both low and high z-scores were associated with preterm birth at 32 and 37 weeks of gestation, however, the effect of preterm birth observed with low weight gain was significantly weaker than that observed using total weight gain [21]. In our study, we observed similar associations of low and high GWG z-scores with a greater risk of preterm birth in women with underweight BMI, normal BMI, and overweight BMI, while only low GWG z-scores were associated with a higher risk of preterm birth in women with obesity BMI. We speculate that this finding may be due to the small range of o low GWG s-score among obese persons in our study. Using the z-score, Leonard et al. found that low and high weight gain was associated with an increased risk of preterm birth and that the optimal range of weight gain with minimal risk of preterm birth decreased with increasing severity of pre-pregnancy overweight/obesity [22]. The different association also informs healthcare planning and commissioning of services, as the level of GWG required to prevent adverse outcomes associated with preterm birth will differ according to BMI classification.

Postdate pregnancy represents a circumstance in which labor does not occur within the physiological term of gestation; this prolongation suggests an alteration in the physiological processes regulating the onset of labor, thus representing a potential risk factor for the fetus [6]. In addition to the relationship between pre-pregnancy BMI and post-term birth [23], we found that a low GWG z-score was associated with an increased risk of post-term birth, nevertheless, higher GWG z-scores were related to a decreased risk of post-term birth. A retrospective cohort study of term, singleton births has demonstrated that GWG increases the risk of a post-term delivery [24]. Denison et al. reported that a greater increase in maternal BMI between the first and third trimesters was also associated with longer gestation [25]. Although our findings indicated that elevated GWG z-scores were associated with a decreased risk of post-term birth, excessive GWG is both associated with an increased risk of complications during pregnancy and childbirth [26]. Thereby, when considering appropriate GWG to reduce the risk of post-term delivery, other adverse outcomes caused by GWG need to be taken into account. Moreover, in this study, low GWG z-score and higher GWG z-scores were associated with both preterm birth and post-term birth. Further studies to estimate the association between the range of perceived ideal GWG with fewer pregnancy outcomes are needed.

This study was a longitudinal-based research study and a larger sample frame that can better understand and seek the association between GWG and preterm birth and post-term birth. However, this study has some limitations. The main limitation is the retrospective nature of this study as the collected data depended entirely on the available data. In addition, although multivariable analyses were utilized to minimize the effect of confounders, potentially unknown or unidentified confounders may exist. Thereby, the associations between GWG and preterm birth, post-term birth, and an adverse outcome combining preterm birth and post-term birth need further studies.

## Conclusions

GWG was associated with preterm and post-term birth outcomes. In clinical practice, pregnant women should be guided to have a clear understanding of weight gain, regular check-ups during pregnancy, reasonable weight control, and reducing the risk of adverse neonatal outcomes from preterm and post-term birth.

## Supplementary Information

**Additional file 1.**

**Additional file 2.**

## Data Availability

The datasets used and/or analyzed during the current study are available from the NVSS database, https://www.cdc.gov/nchs/nvss/births.htm.

## Abbreviations

GWG: Gestational weight gain
BMI: Body mass index
NVSS: National Vital Statistics System
CDC: Centers for Disease Control and Prevention
WIC: Women, infants, and children
GED: General educational development
SD: Standard deviation
ANOVA: Analysis of variance
RCS: Restricted cubic spline

## References

[1] Blencowe H, Cousens S, Oestergaard MZ, Chou D, Moller AB, Narwal R (2012). National, regional, and worldwide estimates of preterm birth rates in the year 2010 with time trends since 1990 for selected countries: a systematic analysis and implications. Lancet (London, England).

[2] Li H, Huang Q, Liu Y, Garmire LX (2020). Single cell transcriptome research in human placenta. Reproduction (Cambridge, England).

[3] Practice bulletin no (2014). 146: Management of late-term and postterm pregnancies. Obstet Gynecol.

[4] Pierrat V, Marchand-Martin L, Arnaud C, Kaminski M, Resche-Rigon M, Lebeaux C (2017). Neurodevelopmental outcome at 2 years for preterm children born at 22 to 34 weeks' gestation in France in 2011: EPIPAGE-2 cohort study. BMJ (Clinical research ed).

[5] Norman JE, Norrie J, MacLennan G, Cooper D, Whyte S, Chowdhry S (2021). Evaluation of the Arabin cervical pessary for prevention of preterm birth in women with a twin pregnancy and short cervix (STOPPIT-2): An open-label randomised trial and updated meta-analysis. PLoS Med.

[6] Coyle K, Quan AML, Wilson LA, Hawken S, Bota AB, Coyle D (2021). Cost-effectiveness of a gestational age metabolic algorithm for preterm and small-for-gestational-age classification. American journal of obstetrics & gynecology MFM.

[7] Zhou YB, Liu JM (2021). Optimal gestational weight gain. The Lancet regional health Western Pacific.

[8] Gaillard R (2015). Maternal obesity during pregnancy and cardiovascular development and disease in the offspring. Eur J Epidemiol.

[9] Goldstein RF, Abell SK, Ranasinha S, Misso ML, Boyle JA, Harrison CL (2018). Gestational weight gain across continents and ethnicity: systematic review and meta-analysis of maternal and infant outcomes in more than one million women. BMC Med.

[10] Champion ML, Harper LM (2020). Gestational Weight Gain: Update on Outcomes and Interventions. Curr DiabRep.

[11] Simko M, Totka A, Vondrova D, Samohyl M, Jurkovicova J, Trnka M (2019). Maternal Body Mass Index and Gestational Weight Gain and Their Association with Pregnancy Complications and Perinatal Conditions. Int J Environ Res Public Health.

[12] Foratori-Junior GA, Jesuino BG, Caracho RA, Orenha ES, Groppo FC, Sales-Peres SHC (2020). Association between excessive maternal weight, periodontitis during the third trimester of pregnancy, and infants' health at birth. Journal of applied oral science : revista FOB.

[13] Kominiarek MA, Saade G, Mele L, Bailit J, Reddy UM, Wapner RJ (2018). Association Between Gestational Weight Gain and Perinatal Outcomes. Obstet Gynecol.

[14] Heusschen L, Krabbendam I, van der Velde JM, Deden LN, Aarts EO, Merién AER (2021). A Matter of Timing-Pregnancy After Bariatric Surgery. Obes Surg.

[15] Liu B, Xu G, Sun Y, Du Y, Gao R, Snetselaar LG (2019). Association between maternal pre-pregnancy obesity and preterm birth according to maternal age and race or ethnicity: a population-based study. Lancet Diabetes Endocrinol.

[16] Hutcheon JA, Bodnar LM (2018). Good Practices for Observational Studies of Maternal Weight and Weight Gain in Pregnancy. Paediatr Perinat Epidemiol.

[17] Arach AAO, Tumwine JK, Nakasujja N, Ndeezi G, Kiguli J, Mukunya D (2021). Perinatal death in Northern Uganda: incidence and risk factors in a community-based prospective cohort study. Glob Health Action.

[18] Bastola K, Koponen P, Härkänen T, Luoto R, Gissler M, Kinnunen TI (2020). Pregnancy complications in women of Russian, Somali, and Kurdish origin and women in the general population in Finland. Womens Health (Lond Engl).

[19] Zielinska MA, Rust P, Masztalerz-Kozubek D, Bichler J, Hamułka J (2019). Factors Influencing the Age of Complementary Feeding-A Cross-Sectional Study from Two European Countries. Int J Environ Res Public Health.

[20] Santos S, Voerman E, Amiano P, Barros H, Beilin LJ, Bergström A, et al. Impact of maternal body mass index and gestational weight gain on pregnancy complications: an individual participant data meta-analysis of European, North American and Australian cohorts. BJOG. 2019; 126(8): 984–995. 10.1111/1471-0528.15661 PMC6554069 30786138

[21] Bodnar LM, Hutcheon JA, Parisi SM, Pugh SJ, Abrams B (2015). Comparison of gestational weight gain z-scores and traditional weight gain measures in relation to perinatal outcomes. Paediatr Perinat Epidemiol.

[22] Leonard SA, Hutcheon JA, Bodnar LM, Petito LC, Abrams B. Gestational Weight Gain-for-Gestational Age Z-Score Charts Applied across U.S. Populations. Paediatric Perinatal Epidemiol. 2018;32(2):161–171. 10.1111/ppe.12435 PMC6637422 29281119

[23] Qazi Q, Liaqat N, Hussain SS, Syed W (2022). Association of high Body Mass Index and postdates pregnancy. Pakistan journal of medical sciences.

[24] Halloran DR, Cheng YW, Wall TC, Macones GA, Caughey AB (2012). Effect of maternal weight on postterm delivery. J Perinatol: official journal of the California Perinatal Association.

[25] Denison FC, Price J, Graham C, Wild S, Liston WA (2008). Maternal obesity, length of gestation, risk of postdates pregnancy and spontaneous onset of labour at term. BJOG : an international journal of obstetrics and gynaecology.

[26] Goldstein RF, Abell SK, Ranasinha S, Misso M, Boyle JA, Black MH (2017). Association of Gestational Weight Gain With Maternal and Infant Outcomes: A Systematic Review and Meta-analysis. JAMA.

