# Supplementary material for: Association between gestational weight gain and preterm birth and post-term birth: a longitudinal study from the National Vital Statistics System database
Source: BMC Pediatr. 2023 Mar 20;23:127. doi: 10.1186/s12887-023-03951-0 (PMC10026488; doi:10.1186/s12887-023-03951-0)
Supplement: Supplementary file 1 — Additional file 1. [file 12887_2023_3951_MOESM1_ESM.docx]

**Supplementary Table 1 The maternal GWG ranges for different gestational ages based on the calculation of z-scores**

| Gestational age | Mean | SD | Recommended range for weight gain |
| --- | --- | --- | --- |
| 24 | 8.4 | 3.9 | 5.61-6.93 |
| 25 | 9.1 | 4 | 6.12-7.60 |
| 26 | 9.7 | 4.1 | 6.57-8.19 |
| 27 | 10.3 | 4.2 | 7.03-8.78 |
| 28 | 11 | 4.3 | 7.55-9.48 |
| 29 | 11.6 | 4.4 | 8.02-10.10 |
| 30 | 12.1 | 4.5 | 8.42-10.64 |
| 31 | 12.6 | 4.6 | 8.82-11.18 |
| 32 | 13.1 | 4.7 | 9.23-11.74 |
| 33 | 13.6 | 4.8 | 9.64-12.30 |
| 34 | 14.1 | 4.9 | 10.05-12.87 |
| 35 | 14.8 | 5.1 | 10.67-13.75 |
| 36 | 15.5 | 5.2 | 11.24-14.52 |
| 37 | 16.1 | 5.3 | 11.73-15.21 |
| 38 | 17 | 5.4 | 12.46-16.19 |
| 39 | 17.7 | 5.6 | 13.10-17.13 |
| 40 | 18.5 | 5.7 | 13.76-18.04 |
| 41/41+ | 19.1 | 5.8 | 14.28-18.77 |

Notes: GWG: gestational weight gain; M: Median; SD: standard deviation.
