# Supplementary figures and images for: Association between gestational weight gain and preterm birth and post-term birth: a longitudinal study from the National Vital Statistics System database
Source: BMC Pediatr. 2023 Mar 20;23:127. doi: 10.1186/s12887-023-03951-0 (PMC10026488; doi:10.1186/s12887-023-03951-0)

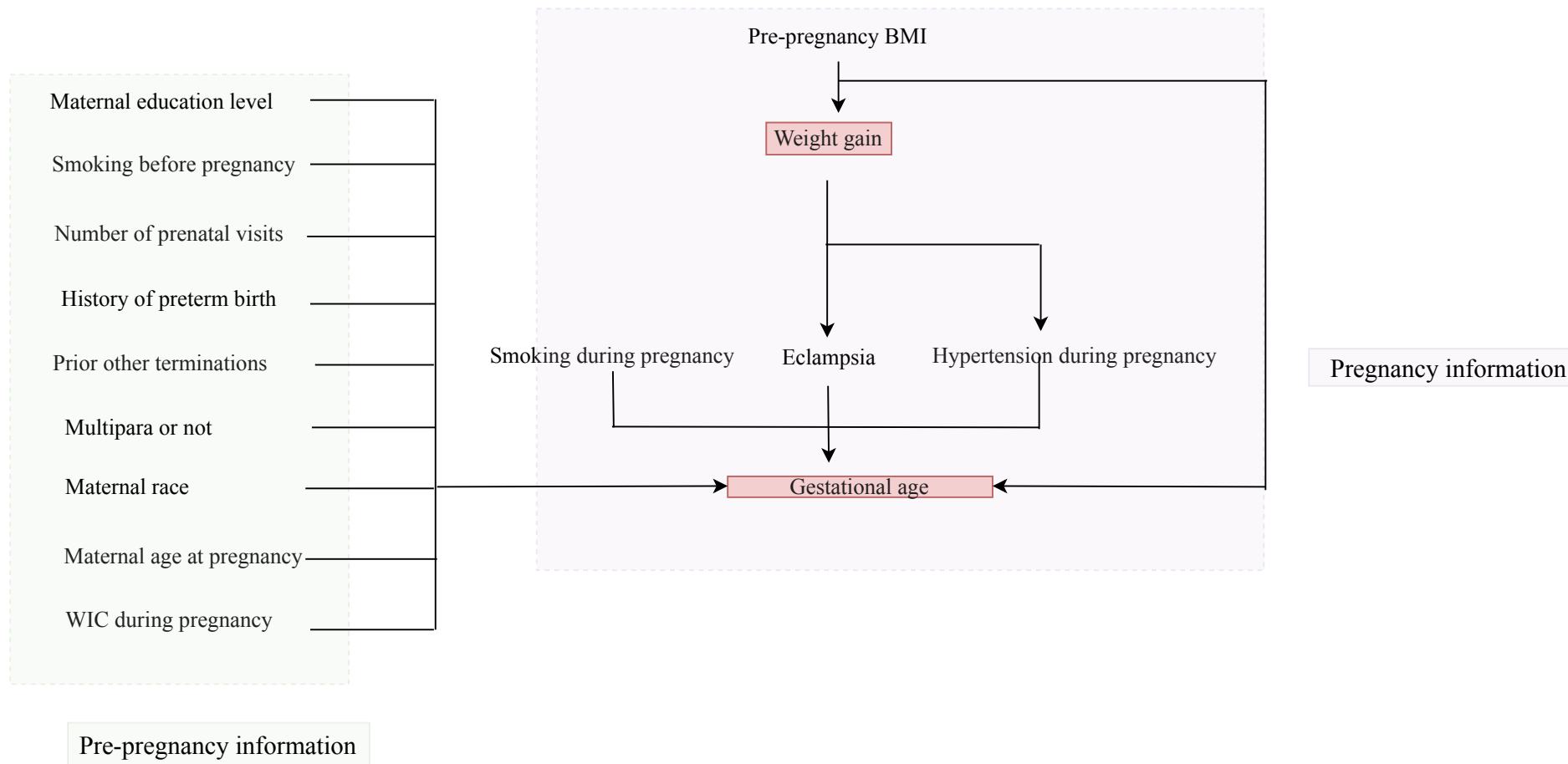

Supplementary Figure 1 DAG of the potential confounders screening

Supplement: Supplementary file 2 — Additional file 2. [file 12887_2023_3951_MOESM2_ESM.pdf]
